# A Randomized Controlled Trial of Lisinopril to Decrease Lymphoid Fibrosis in Antiretroviral-Treated, HIV-infected Individuals

**DOI:** 10.20411/pai.v2i3.207

**Published:** 2017-08-02

**Authors:** Leslie R. Cockerham, Steven A. Yukl, Kara Harvill, Ma Somsouk, Sunil K. Joshi, Elizabeth Sinclair, Teri Liegler, Rebecca Hoh, Sophie Lyons, Peter W. Hunt, Adam Rupert, Irini Sereti, David R. Morcock, Ajantha Rhodes, Claire Emson, Marc K. Hellerstein, Jacob D. Estes, Sharon Lewin, Steven G. Deeks, Hiroyu Hatano

**Affiliations:** 1 Division of Infectious Diseases, Medical College of Wisconsin, Milwaukee, Wisconsin; 2 Department of Medicine, San Francisco VA Medical Center, and University of California, San Francisco (UCSF), San Francisco, California; 3 HIV, Infectious Diseases, and Global Medicine Division, San Francisco General Hospital, University of California, San Francisco, California; 4 Division of Gastroenterology, San Francisco General Hospital, University of California, San Francisco, California; 5 National Institute of Allergy and Infectious Diseases, National Institutes of Health, Bethesda, Maryland; 6 Frederick National Laboratory, Leidos Biomedical Research, Frederick, Maryland; 7 Department of Infectious Diseases, The Alfred Hospital and Monash University, Melbourne, Victoria, Australia; 8 Doherty Institute for Infection and Immunity, University of Melbourne, Melbourne, Victoria, Australia; 9 Kinemed, Inc., Emeryville, California; 10 Department of Nutritional Science and Toxicology, University of California, Berkeley, California

**Keywords:** Anti-Inflammatory Agents/*therapeutic use, CD4 Lymphocyte Count, Disease Reservoirs/*virology, HIV, Immunology, Lymphoid fibrosis, T-cell activation

## Abstract

**Background:**

In HIV infection, lymphoid tissue is disrupted by fibrosis. Angiotensin converting enzyme inhibitors have anti-fibrotic properties. We completed a pilot study to assess whether the addition of lisinopril to antiretroviral therapy (ART) reverses fibrosis of gut tissue, and whether this leads to reduction of HIV RNA and DNA levels.

**Methods:**

Thirty HIV-infected individuals on ART were randomized to lisinopril at 20mg daily or matching placebo for 24 weeks. All participants underwent rectal biopsies prior to starting the study drug and at 22 weeks, and there were regular blood draws. The primary end point was the change in HIV RNA and DNA levels in rectal tissue. Secondary outcomes included the change in 1) HIV levels in blood; 2) Gag-specific T-cell responses; 3) levels of T-cell activation; and 4) collagen deposition.

**Results:**

The addition of lisinopril did not have a significant effect on the levels of HIV RNA or DNA in gut tissue or blood, Gag-specific responses, or levels of T-cell activation. Lisinopril also did not have a significant impact on lymphoid fibrosis in the rectum, as assessed by quantitative histology or heavy water labeling.

**Conclusions:**

Treatment with lisinopril for 24 weeks in HIV-infected adults did not have an effect on lymphoid fibrosis, immune activation, or gut tissue viral reservoirs. Further study is needed to see if other anti-fibrotic agents may be useful in reversing lymphoid fibrosis and reducing HIV levels.

**Clinical Trials Registration:**

NCT01535235

## INTRODUCTION

An important feature of the pathophysiology of HIV infection is the disruption of the lymphoid architecture by collagen deposition [1]. This resultant fibrosis leads to damage of the fibroblastic reticular cell network (FRCn). The FRCn is crucial for the delivery of cytokines and growth factors (such as IL-7) that promote survival and homeostatic proliferation of T cells [2–4]. Fibrosis of lymphoid tissues persists despite antiretroviral therapy (ART) [5–7]. Furthermore, collagen deposition is inversely associated with CD4^+^ T-cell reconstitution in lymphoid tissue [1, 6, 8], and the amount of collagen deposition before ART predicts the magnitude of peripheral T-cell recovery after ART initiation [9–11].

Collagen deposition is linked to chronic immune activation and inflammation. In an effort to regulate the immune response, T-regulatory cells are recruited to lymphoid tissues and produce transforming growth factor β (TGF-β), which stimulates collagen production [3, 12, 13]. Previous studies have shown both a spatial and temporal association between TGF-β expression and collagen deposition [12]. This process occurs rapidly after HIV infection and is progressive. Not only does this fibrosis disrupt the FRCn and T-cell homeostasis, but it may also prevent the effective interaction between Antigen Presenting Cells (APCs) and cytotoxic T lymphocytes and could be a factor in the ineffective clearance of HIV-infected cells [2, 4, 14]. However, collagen deposition has been shown to be a dynamic process that can be reversed [6, 15]. Therefore, it may be a modifiable barrier to restoring normal immune function and improving immune clearance of the viral reservoir.

Multiple studies have shown that angiotensin converting enzyme (ACE) inhibitors have anti-fibrotic properties through inhibition of TGF-β1 [16–19]. Due to the long clinical history of safely using ACE-inhibitors, and due to its putative anti-fibrotic potential, we conducted a randomized, double-blind placebo-controlled trial to examine whether addition of the ACE-inhibitor lisinopril decreases collagen deposition in the gut tissue and ultimately decreases the amount of HIV RNA and DNA in HIV-infected individuals receiving suppressive ART. As the degree of lymphoid fibrosis appears to be a strong determinant of T-cell recovery [8, 9] we enriched our study with individuals who failed to normalize their CD4^+^ T-cell counts with suppressive ART [20]. We hypothesized that treatment with lisinopril would reduce lymphoid fibrosis in the gut, improve HIV specific responses, and decrease the levels of HIV RNA and DNA.

## METHODS

### Study Participants

Thirty-one HIV-infected individuals on suppressive ART (HIV VL < 40-75 copies/mL) for ≥1 year were enrolled in this randomized, placebo-controlled study (clinical trials registration NCT01535235). Enrollment was stratified by CD4^+^ T-cell counts of < 350 cells/μL (immunologic non-responders, INRs) and ≥ 350 cells/μL (immunologic responders, IRs) (see Consort diagram in supplementary materials). Exclusion criteria included a known history of diabetes mellitus, cardiovascular disease, or collagen vascular disease. Individuals with a serum creatinine of > 1.5 mg/dL or who were already taking an ACE-inhibitor or angiotensin receptor blocker were also excluded. Study personnel and all authors were masked to the study group assignment until data collection and analyses were completed. Adherence to the study drug was measured at study visits by self-report and by pill count. All participants underwent sigmoidoscopy with rectal biopsies prior to starting the study drug and at 22 weeks, and there were frequent blood draws. All participants provided written informed consent. This study was approved by the University of California, San Francisco (UCSF) Committee on Human Research. An independent Data Monitoring Committee comprising 3 independent individuals from the scientific community met at 12 and 36 weeks after the enrollment of the first subject and at 36 weeks after the enrollment of the last subject.

## HIV RNA/DNA MEASUREMENTS

HIV RNA and DNA were measured in tissue from rectal biopsies prior to starting the study drug and again at week 22. Rectal biopsies were processed using a previously published method of collagenase digestion and mechanical disruption [21]. Three replicates of 500 ng of DNA and RNA were assayed for total HIV DNA and RNA transcripts, respectively, using a published quantitative (q) PCR assay that uses primers and probe from the LTR region [22]. External standards were prepared for both DNA and RNA [23, 24]. The HIV DNA copy numbers were normalized to cellular input into the PCR as determined by DNA mass, and the cell equivalents were confirmed by qPCR for TERT [22]. The HIV RNA copy numbers were normalized to cellular input as determined by RNA mass and by qPCR for GAPDH [22].

Levels of cell-associated HIV RNA (CA-RNA) and DNA were measured from CD4^+^ T cells isolated from cryopreserved PBMCs at baseline, week 4, and week 24. The CD4^+^ T cells were isolated using an isolation kit (Stemcell Technologies, Vancouver, Canada; purity 97%), and RNA and DNA were extracted (Allprep isolation kit, Qiagen, Valencia, CA). For quantification of CA-RNA, a semi-nested real time q PCR was used with a first round amplification of 15 cycles, as previously described [25]. The second round used primers to gag [26]. The HIV RNA copy numbers were standardized to cellular equivalents using an 18s TaqMan gene expression assay (Thermo Fisher, Waltham, MA). The lower limit of detection for CA-RNA was 1 copy per well, and PCR amplification of cDNA for CA-RNA was performed in quadruplicate with an intra-assay coefficient of variation (CV) of 32%. The HIV DNA was quantified as previously described [27], and PCR for HIV DNA was performed in triplicate for all samples with an intra-assay CV of 21%. In all assays, a control without reverse transcriptase was used. Full details are available in the Supplementary Materials.

### T-Cell Immunophenotyping and Cytokine Flow Cytometry

Immunophenotyping was performed on cryopreserved PBMCs and fresh rectal mucosal cells to measure T-cell activation and the frequency of Gag-specific CD4^+^ and CD8^+^ T cells as previously described [28, 29] The frequency of HIV-specific T cells, in PBMCs and isolated rectal mucosal cells, was measured by cytokine flow cytometry (CFC) as previously described [30, 31]. For full details of the immunophenoptyping and CFC see the Supplementary Materials.

### Markers of Monocyte Activation

Several biomarkers of inflammation and innate immune activation have been associated with HIV disease progression and mortality [32–35]. These systemic inflammatory processes are thought to contribute to fibrosis and may also result in the production of pro-inflammatory molecules by activated cells, such as intestinal myofibroblasts [10, 36]. To determine whether treatment with lisinopril might alter this inflammatory milieu, we measured levels of markers of inflammation (interleukin 6 [IL-6]), coagulation (D-dimer), monocyte activation (soluble CD14 [sCD14], soluble tumor necrosis factor α receptor I and II (sTNF-RI, sTNF-RII), and fibrosis (hyaluronic acid [HA]) from cryopreserved plasma specimens from the baseline visit, week 4, and week 24 visits. Plasma samples were analyzed for D-dimer concentrations using an ELFA (Enzyme Linked Fluorescent Assay) on a VIDAS instrument (bioMerieux Inc., Durham, NC). Determinations of IL-6 were performed using an electrochemiluminescense ELISA (Meso Scale Discovery, Gaithersburg, MD). Soluble CD14 and tissue factor analysis was performed using a standard ELISA (R&D Systems, Minneapolis, MN). All the tests listed were performed according to the manufacturer's instructions.

### Immunohistochemistry and Quantitative Image Analysis

Immunohistochemical staining was performed for collagen 1, myeloperoxidase positive PMNs (a marker of epithelial damage), Phospo-Smad3, and CD4^+^ T cells in the rectal biopsy tissue, and quantitative image analysis was performed as previously described [37, 38] and in the Supplementary Materials.

### Measurement of Collagen Synthesis Rate Using ^2^H_2_O Heavy Water Labeling

We also investigated the use of ^2^H_2_O (heavy water) to quantify the rate of new collagen deposition in rectal tissue in treated HIV-infected individuals. The first 18 individuals enrolled in this study received outpatient oral doses of ^2^H_2_O for 4 weeks prior to each colorectal biopsy. Then ^2^H_2_O enrichment in total body water was quantified from weekly salivary swabs during ^2^H_2_O administration. Single 3 mm rectal biopsy pieces were subjected to sequential physical and chemical extraction methods to fractionate the extracellular matrix based on solubility in guanidine HCl [39]. Guanidine-soluble collagen represents more recently synthesized, less cross-linked collagen, while guanidine-insoluble collagen represents more mature cross-linked collagen [39]. Incorporation of the ^2^H_2_O tracer into collagen in rectal tissue was quantified by liquid-chromatography-tandem mass spectrometry and the fractional synthesis rate (FSR, per week) was calculated for guanidine-soluble and guanidine-insoluble collagen, as previously described [39–41].

## STATISTICAL ANALYSIS

The primary end point was the change in HIV RNA and DNA levels in gut tissue. Inputs for detectable effect calculations came from a previous study by our group [42]. In that study, the mean baseline GALT RNA was 53.3 copies per million rectal mononuclear cells (RMCs), with a residual standard deviation (SD) of 21.4 and within-subject correlation of 0.89. Based on these data, we estimated that the sample size of 15 treated and 15 control subjects would provide 80% power to detect a difference as small as 10.4 copies per million RMCs, or 20% of the baseline mean. Similarly, mean baseline GALT DNA was 3288 copies per million RMCs, with a residual SD of 841 and within-subject correlation of 0.90. The sample size of 15 treated and 15 control subjects provided 80% power to detect a difference of 398 copies per million RMCs, or 12% of the baseline mean. These calculations accounted for a loss to follow-up (or unusable data) of 5% of subjects, as well as within-subject correlation of repeated observations.

Secondary outcomes included the change in 1) HIV RNA and DNA levels in PBMCs; 2) CD4^+^ and CD8^+^ total Gag-specific responses (IFN-γ, IL-2, TNF-α, or CD107a) in rectal tissue and PBMCs; 3) the percentage of CD38^+^HLA-DR^+^ CD4^+^ and CD8^+^ T cells in rectal tissue and PBMCs; and 4) the rates of collagen turnover and amount of collagen deposition in rectal tissue. Levels were compared using the Wilcoxon rank sum test or the Wilcoxon signed rank test for paired samples. Linear mixed effects models with random intercepts and slopes were used to evaluate the association of the treatment group and responder status with the rates of change in HIV RNA and DNA levels in PBMCs, as well as T-cell activation and biomarker levels in PBMCs. Interaction terms between treatment group and time were used to determine whether the rate of change in these markers differed between the lisinopril-treated and placebo groups. All statistical analyses were performed using STATA/SE 12 (Stata Corp, College Station, TX).

## RESULTS

### Baseline Characteristics

Thirty-one individuals were enrolled in the study. One participant was withdrawn from the study at week 12 by investigators due to an increase in serum creatinine. Baseline characteristics of the participants were similar (Table 1). The median age of all participants was 54 years (IQR 47–57) and all participants were male. The median CD4^+^ T-cell count was 368 cells/μL (IQR 241–636).

**Table 1:** Baseline Characteristics

	Placebo (n = 15)	Lisinopril (n = 16))	*P*-value
Age (median, IQR)	54 (47-58)	53 (44-57)	0.31
Sex (% Male, n)	100% (15)	100% (15)	
Race (%, n)			
White	60% (9)	88% (14)	
African-American	7% (1)	12% (2)	
Latino/Hispanic	20% (3)		
Asian	7% (1)		
Pacific Islander	7% (1)		
CD4^+^ T cell count (median, IQR)	392 (237–641)	362 (241–435)	0.87
CD8^+^ T cell count (median, IQR)	691 (416–1282)	654 (453–1187)	0.98
Baseline rectal HIV RNA (copies/10^6^ rectal cells)	147 (57–358)	137 (53–447)	0.81
Baseline rectal HIV DNA (copies/10^6^ rectal cells)	181 (80–225)	126 (42–177)	0.41

Data are % (no.) of patients, unless otherwise indicated.

Abbreviations: IQR, interquartile range.

### Adverse Events

No individuals voluntarily discontinued the study due to adverse events. Two individuals noted a mild dry cough, but continued study participation and the cough resolved in both individuals. One individual was withdrawn from the study due to an increase in serum creatinine at week 12, which had already returned to baseline at the time of discontinuation and was deemed not to be related to the study drug. At 24 weeks, individuals receiving lisinopril had a mean increase of 0.06 mg/dL in serum creatinine compared to the placebo group, which had stable creatinine levels, although this difference between groups was not statistically significant (placebo: +0.003 mg/dL change, *P* = 0.08).

### Cell-Associated HIV RNA and Proviral DNA

**Figure 1.**
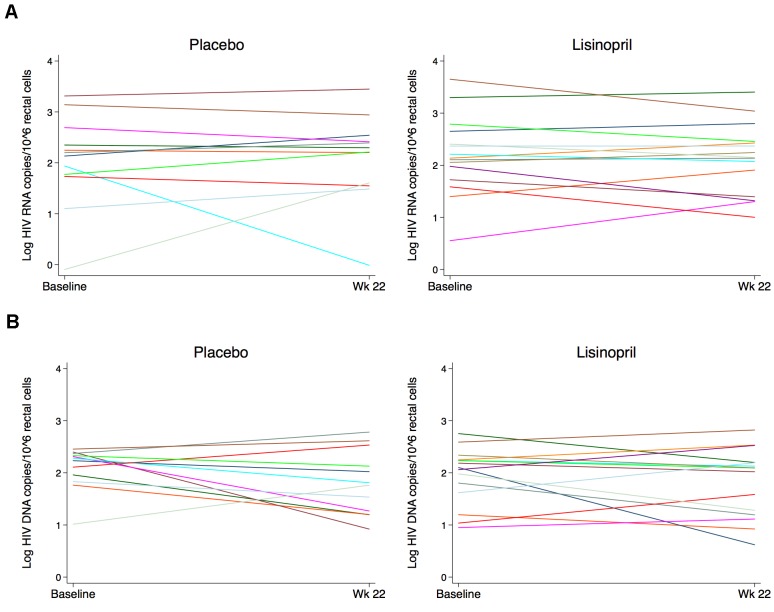
Change in Log HIV RNA copies/per 10^6^ rectal cells (A) and Log HIV DNA copies/per 10^6^ rectal cells (B) from baseline to Week 22 in the placebo and lisinopril treatment groups. Each line represents an individual participant.

**Figure 2.**
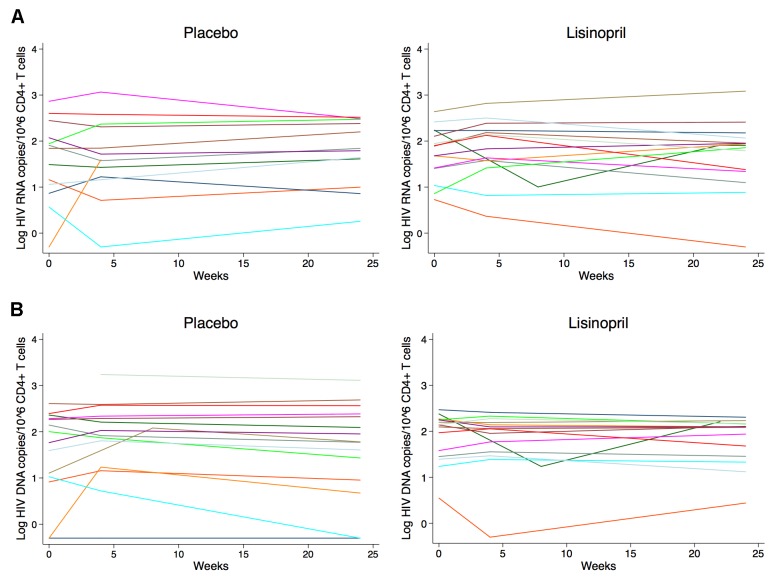
Change in Log HIV RNA copies/per 10^6^ CD4^+^ T cells (A) and Log HIV DNA copies copies/per 10^6^ CD4^+^ T cells from Week 0 to Week 24 in the placebo and lisinopril treatment groups.

Baseline median CA-HIV RNA levels in rectal cells were similar between placebo and lisinopril groups at 147 (IQR 57–358) copies and 137 (IQR 53–447) copies/million gut cells, respectively. Total proviral HIV DNA levels in the gut were higher at baseline in the placebo group compared to the lisinopril-treated group, but this was not statistically significant (181 vs 126 copies/million gut cells, *P* = 0.41). Baseline median levels of CA-HIV RNA in the gut were higher among IRs compared to INRs (201 vs 59 copies/million gut cells, *P* = 0.02), but this was primarily driven by a single individual with a high HIV RNA level of > 4000 copies/million. Baseline levels of HIV DNA were more similar between the 2 responder groups (126 vs 172 copies/million gut cells, *P* = 0.16). Treatment with lisinopril did not affect the change in CA-RNA or proviral DNA in rectal cells (Figure 1), and CA-RNA and DNA were also similar in CD4^+^ T cells isolated from PBMCs between treatment groups both at baseline and after 24 weeks of treatment with study drug (Figure 2). Similarly, no difference was noted in the rate of change of HIV DNA or RNA over time between treatment groups using a mixed effects model. There was also no difference in response to treatment by immunologic responder status.

### T-Cell Activation and HIV-specific Responses

**Figure 3.**
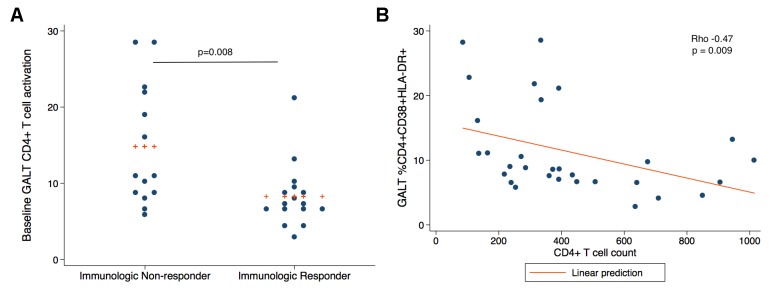
(A) Baseline levels of CD4^+^ T cell activation in rectal cells in immunologic non-responders and immunologic responders. (B) The correlation between the percentage of CD4^+^CD38^+^DR^+^ T cells in the rectal lymphoid tissue and the peripheral CD4^+^ T cell count. A linear prediction line is shown in red.

There was no difference in the percentage of CD38^+^DR^+^ CD4^+^ and CD8^+^ T cells between treatment groups at baseline. However, INRs had elevated median levels of CD38^+^HLA-DR^+^ CD4^+^ and CD8^+^ T cells in rectal cells at baseline compared to IRs (CD4: median 11.1% vs 7.23%, *P* = 0.008, CD8: 24.0% vs 18.4%, *P* = 0.09) (Figure 3A) and this correlated negatively with the peripheral CD4^+^ T cell count (rho = -0.47, *P* = 0.009) (Figure 3B). The T-cell activation in PBMCs at baseline was also negatively correlated with peripheral CD4^+^ T cell count (% CD4^+^CD38^+^HLA-DR^+^, rho -0.57, *P* = 0.001; % CD4^+^PD-1^+^, rho = -0.66, *P* = 0.0001), as has been seen previously in other studies [43]. The addition of lisinopril did not have a significant effect on the change in CD4^+^ and CD8^+^ T- cell activation or Gag-specific responses over time (data not shown).

### Markers of Inflammation and Monocyte Activation

We measured levels of IL-6, D-dimer, sCD14, sTNF-RI, sTNF-RII and HA from cryopreserved plasma specimens from the baseline visit, week 4 and week 24 visits. At baseline, several biomarkers of inflammation and coagulation were independently associated with demographic and clinical factors such as age, baseline CD4^+^ T-cell count, and levels of T-cell activation (Supplemental Table 1). These covariates were included in linear mixed effects modeling when appropriate. However, there was no difference in the levels of IL-6, D-dimer, sCD14, sTNF-RI, sTNF-RII, or HA over time between treatment groups using a mixed effects model (data not shown).

### Measures of collagen deposition

**Figure 4.**
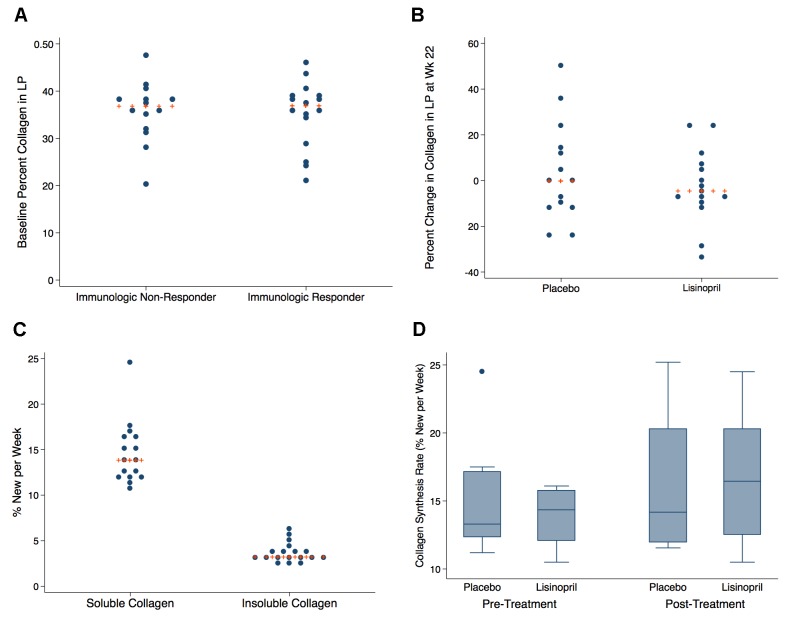
(A) Baseline percent collagen deposition by immune responder status. Plus signs denote the median. (B) Percent change in collagen deposition in the lamina propia (LP) at week 22. (C) Baseline collagen synthesis rates (percent new per week) of guanidine-soluble and guanidine-insoluble collagen in all participants measured by heavy water labeling. (D) Change in collagen synthesis rates (percent new per week) of guanidine-soluble collagen in the placebo and lisinopril groups at baseline and at week 22.

We used immunohistochemistry and quantitative image analysis to determine whether administration of lisinopril reduced the abundance of collagen I deposition in the lamina propia (LP), and when possible the follicular aggregates (FA) (n = 8), from rectal biopsies. At baseline, there was no correlation between the percentage of the area that stained positive for collagen 1 in the LP or FA of rectal biopsies and covariates such as age, CD4^+^ or CD8^+^ T-cell counts or T-cell activation levels (data not shown). The IRs and INRs had similar percentages of collagen 1 deposition in the LP (37% and 37% respectively, *P* = 0.99) (Figure 4A) and FA (21% and 15%, *P* = 0.46) at baseline. In the LP, a median decrease in the percent change of collagen deposition of 4.7% (IQR -8.9% –6.3%) was seen in the lisinopril group at week 22 compared to a median decrease of 0.4% in the placebo group (IQR -12.1%–15.4%), however this change was not statistically significant (*P* = 0.63) (Figure 4B). Decreases in collagen deposition in the FA of both the placebo and lisinopril groups were seen, but were not significantly different (-12.8% and -17.8% respectively, *P* = 0.88).

A total of 18 individuals underwent ^2^H_2_O labeling to measure collagen synthesis rates—10 participants in the placebo group and 8 in the lisinopril group. Baseline levels of guanidine-insoluble and guanidine-soluble collagen did not differ significantly between groups or between IR and INR status (data not shown). Active collagen synthesis was measurable in both the guanidine-soluble and guanidine-insoluble fractions. At baseline there was a significant difference in the baseline median FSR of guanidine-insoluble collagen compared to guanidine-soluble in rectal tissue. The FSR of guanidine-soluble collagen in rectal tissue was much higher than guanidine-insoluble collagen, with a median FSR of 12.8% per week (IQR 12.3%–16.1%) compared to an FSR of 3.2% per week (IQR 2.9%–3.9%) in guanidine-insoluble collagen (Figure 4C). The calculated half-lives of the collagen pools (assuming a steady-state in collagen pool size) is approximately 5 weeks for guanidine-soluble and approximately 23 weeks for guanidine-insoluble pools. Median collagen synthesis rates in rectal tissue did not differ significantly between the 2 treatment arms at baseline (placebo 13.3% vs 14.5%, *P* = 0.56) or at the end of treatment (14.2% vs 16.5%, *P* = 0.96) (Figure 4D). Similarly to the immunohistochemistry findings, there was no association noted between baseline collagen FSRs and variables such as age, CD4^+^ T-cell counts, T-cell activation levels, or the level of collagen deposition (data not shown).

### Gut damage and CD4+ T cell recovery

**Figure 5.**
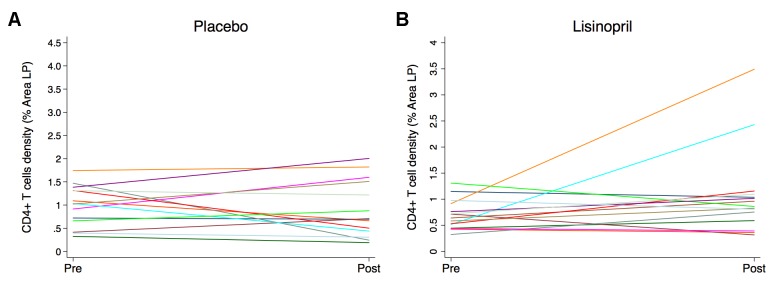
Change in density of CD4^+^ T cells as a percentage of area in the lamina propia from baseline to week 22 in the placebo and lisinopril treatment groups.

We used immunohistochemistry and quantitative image analysis to determine whether administration of lisinopril would improve the reconstitution of CD4^+^ T-cell populations in the LP or FA, the extent of infiltration of myeloperoxidase positive PMNs, or the levels of TGF-β–dependent pSmad3 signaling, in rectal tissue. The median density of CD4^+^ T cells within the LP was higher in the placebo group compared to the lisinopril-treated group at baseline (1.02% vs 0.64%, *P* = 0.08) and increased during treatment in the lisinopril group compared to no change in the placebo group (0.24% vs -0.05%, *P* = 0.22) (Figure 5), although this was partially driven by 2 individuals. Neither the lisinopril nor placebo group had any change in CD4^+^ T cells in the FA; however, a reduction in the density of macrophages in the LP was noted in the placebo group with no change in the lisinopril group (data not shown). There were no differences in the percentage of myeloperoxidase positive PMNs or levels of pSMAD3 signaling between groups in either the LP or FA (data not shown).

## DISCUSSION

In this randomized, double-blind, placebo-controlled study, we found that the addition of lisinopril to a suppressive antiretroviral regimen did not have a significant impact on lymphoid fibrosis or fibrogenesis rate in the rectum, as assessed by quantitative histology or ^2^H_2_O labeling, respectively. Furthermore, we did not find any significant change in HIV DNA and RNA levels, T-cell and monocyte activation, or HIV-specific responses in either rectal tissue or blood over time.

Although we hypothesized that those individuals with poor immunologic recovery after starting ART might have increased levels of fibrosis at baseline and respond differently to an anti-fibrotic agent, we did not observe this.

Multiple studies have demonstrated elevated levels of TGF-β in HIV infection and a relationship between increasing lymphoid fibrosis and disease progression and poor immunologic recovery in treated HIV. Furthermore, the role of TGF-β mediated fibrosis is also being examined in HIV-associated cardiovascular disease [44]. This is an active area of investigation and an agent which reverses fibrosis could be expected to have favorable effects on immune function, disease progression, and potentially even comorbidities.

We chose lisinopril based on *in vitro* and *in vivo* data showing an ability of ACE-inhibitors to reduce TGF-β levels and resultant fibrosis in other tissues, as well as the record of safety and experience with this drug. However, given that we did not find a difference in collagen turnover or levels in rectal lymphoid tissue and did not see a change in pSMAD 2,3 expression, it may be that lisinopril does not have a similar effect in lymphoid tissues or has inefficient drug penetration in fibrotic tissues. Another possible reason why an effect of lisinopril therapy was not observed could be the small sample size, which is a limitation of this study. We did see an increase in the density of CD4^+^ T cells in the LP in the lisinopril-treated group compared to the placebo group, and it is unclear whether this might have been an early indication of some benefit to treatment with lisinopril. Therefore, increased duration of treatment may be required to see additional benefits including changes in collagen levels.

A recent study in rhesus macaques showed that the anti-fibrotic agent pirfenidone reduced levels of fibrosis in the paracortical T-cell zone of lymph node tissue when started prior to infection and after only 6 weeks of infection [45]. Given that lymphoid fibrosis starts early in HIV infection, anti-fibrotic agents might be most beneficial when started early after infection. All of the individuals in our study were in the stage of chronic HIV infection, and many were later in the disease course with lower peripheral blood CD4^+^ T-cell levels. Nevertheless, data from the Berlin patient, provides hope that effective interventions in chronic disease could result in regression of lymphoid fibrosis [6]. Moreover, our data show for the first time that chronically HIV-infected individuals on suppressive anti-retroviral regimens exhibit active collagen synthesis in rectal tissue. Measured FSR values of approximately 13% replacement/week for the less cross-linked guanidine-soluble pool indicate a half-life of approximately 5–6 weeks. Dynamicity of lymphoid tissue collagen pools is consistent with the possibility of reversal of fibrosis if there were an effective anti-fibrotic therapy. However, chronically infected individuals who have increased levels of collagen deposition may require more prolonged periods of treatment to see effects on fibrosis levels. Again, this may be another reason why we did not see an effect of lisinopril treatment on collagen deposition.

This is the first study, to our knowledge, to look at the effects of a drug intervention with putative anti-fibrotic properties in HIV-infected individuals. Although we did not find an effect of lisinopril therapy on collagen levels or synthesis rates, an anti-fibrotic agent may nonetheless be useful as an adjuvant therapy to restore normal immune function and contribute to the eradication of the latent reservoir. This study does raise the need for further examination of the *in vitro* and in *vivo* effects of anti-fibrotic agents and the identification of biomarkers that might predict regression of fibrosis.

## Figures and Tables

**Supplementary Figure 1.**
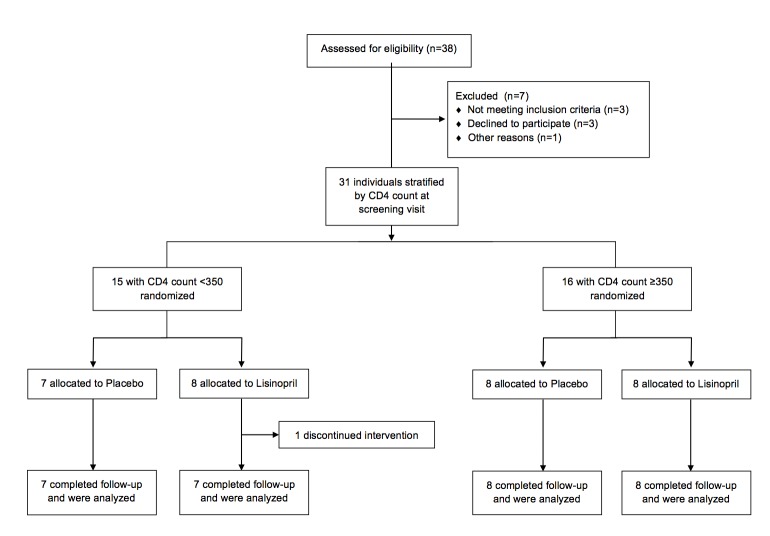
Participant Flow Diagram - Participant Flow Diagram as per the Consolidated Standards of Reporting Trials (CONSORT) Guidelines

## SUPPLEMENTARY METHODS

### HIV RNA/DNA MEASUREMENTS IN RECTAL TISSUE

Rectal biopsies were dissociated using a published method [21]. Briefly, biopsies were subjected to three rounds of collagenase digestion, mechanical disruption (by passing through a blunt 16 gauge needle), clarification (by passing through a 70 μm cell strainer), and washing. The 3 aliquots of strained and washed cells were then combined and counted, and aliquots were stored in RNAlater (Applied Biosystems) at -80C. For nucleic acid extraction, cells were lysed using 1 ml of Qiazol reagent (Qiagen), homogenized using QIAshredder spin columns (Qiagen), and centrifuged at 12,000*g* for 15 minutes at 4°C. RNA was extracted from the aqueous layer using the miRNeasy Mini kit (Qiagen) with on-column DNAase treatment (Qiagen RNase-Free DNase Set) and eluted in RNase-free water. The DNA was extracted from the interphase using 0.5 ml of back extraction buffer (4M guanidine thiocyanate, 50mM Na citrate, and 1M Trizma base), followed by precipitation with 500 μl of isopropanol, 2 washes with 75% ethanol, and resuspension in EB buffer (Qiagen). The RNA and DNA concentrations were measured using a ND-1000 spectrophotometer (NanoDrop).

Three replicate samples of 500 ng of DNA from each donor were assayed for total HIV-1 DNA using a published quantitative real-time PCR (qPCR) assay [22] that uses primers and probe from the LTR region. Primers were F522-43 (5′ GCC TCA ATA AAG CTT GCC TTG A 3′; HXB2 522–543) and R626-43 (5′ GGG CGC CAC TGC TAG AGA 3′; 626–643). The probe, 5′ CCA GAG TCA CAC AAC AGA CGG GCA CA 3′, was dual-labeled with 6-FAM(5′) and Black Hole Quencher BHQ-1(3′). The reaction volume was 50 μl with 25 μl of 2× Gene Expression Master Mix (Applied Biosystems), 10pmol of each primer, 10pmol of probe, and 5 μl of DNA (100 ng/ μl). Cycling conditions were: 50°C for 2 minutes, 95°C for 10 minutes, then 60 cycles of 95°C for 15 seconds and 59°C for 1 minute. External standards were prepared from pNL4-3 DNA, and the number of molecules was calculated by DNA mass, as determined by the ND-1000 spectrophotometer; absolute copy numbers were confirmed by droplet digital PCR [23]. Copy numbers of HIV-1 DNA were normalized to cellular input as determined by DNA mass (assuming 1 μg total DNA corresponds to 160,000 cells). As an alternative method of normalizing to cell numbers, all DNA samples were assayed for TERT by qPCR [22] with the conditions above, except with 2.5 μl of 20× TERT primer/probe mix [Applied Biosystems] and 3 μl of DNA.

Three replicates of up to 500 ng of RNA from each sample were assayed for total processive HIV-1 RNA transcripts using a published qRT-PCR assay [22] that uses primers and probe from the LTR region (as above). The reaction volume was 50 μl with 25 μl of 2× One Step RNA-to-Ct mix (Applied Biosystems), 10pmol of each primer, 10pmol of probe, 1.25 μl of 40× RT (Applied Biosystems), and 5 μl of RNA (100 ng/μl). Cycling conditions were as follows: 48°C for 20 minutes, 95°C for 5 minutes, then 60 cycles of 95°C for 15 seconds and 59°C for 1 minute. External standards (1 to 10^5^) of genomic HIV-1 RNA were prepared from supernatants of NL4-3-infected cells by sequential freeze-thaw (which lyses cells but not virions), nuclease treatment (to digest any free nucleic acids), heat inactivation [24, 46, 47], RNA extraction (using the QIAmp Viral RNA Mini Kit), and then quantification of the RNA by replicate measurements using the Abbot Real Time assay. Copy numbers of HIV-1 RNA were normalized to cellular input into the PCR, as determined by RNA concentration (assuming that 1 ng RNA corresponds to 1000 cells). As an alternative method of normalizing to cell numbers, all RNA samples were assayed for GAPDH by qRT-PCR [22] with the conditions above, except with 2.5 μl of 20× GAPDH primer/probe mix [Applied Biosystems] and 3 μl of RNA.

### HIV RNA/DNA MEASUREMENTS IN CD4^+^ T-CELLS

CD4^+^ T-cells were isolated from stored peripheral blood mononuclear cells (PBMC) using a CD4^+^ T-cell isolation kit (Stemcell Technologies, Vancover Canada; purity 97%) and RNA and DNA extracted (Allprep isolation kit, Qiagen). For quantification of cell-associated HIV RNA (CARNA), a semi-nested qPCR was used with a first round amplification of 15 cycles to ensure that the following second round amplification was in the linear range between 1 to 46,000 input copies, as previously described by Pasternak, *et al* [25]. The second round used primers to gag [26]. Copy numbers of HIV RNA were standardized to cellular equivalents using an 18s TaqMan gene expression assay (Thermo Fisher) The lower limit of detection LLOD for CA-RNA was 1 copy per well. If there was detectable HIV RNA present but < 1 copy per well, this sample was included as 0.5 copies/well. If there was no detectable signal, the sample was designated as zero. The PCR amplification of cDNA for CA-RNA was performed in quadruplicate with an intra-assay coefficient of variation (CV) of 32%. In all assays, a control without reverse transcriptase (RT) was used. If there was any amplification from the no RT control, ie, evidence of DNA contamination, a second stored sample was re-extracted. If contaminating DNA persisted, the reading was excluded. In the 89 samples tested no DNA contamination was detected. The HIV DNA was quantified as previously described [27], and PCR for HIV DNA was performed in triplicate for all samples with an intra-assay CV of 21%. Integrated DNA was measured in total CD4^+^ T-cells as previously described [48].

### T CELL IMMUNOPHENOTYPING AND CYTOKINE FLOW CYTOMETRY

Immunophenotyping and Cytokine Flow Cytometry (CFC) were performed on cryopreserved PBMC and fresh colorectal mucosal cells to measure T-cell activation and the frequency of Gag-specific CD4^+^ and CD8^+^ T cells. Cryopreserved PBMCs were thawed and washed as previously described [49]. Mucosal lymphocytes were isolated from fresh colorectal biopsies by enzymatic digestion and mechanical disruption as previously described [29]. For Immunophenotyping both PBMC and mucosal colorectal cells were stained with LIVE/DEAD® Fixable Aqua Dead Cell Stain Kit (Invitrogen, Carlsbad, CA) to exclude non-viable cells and then stained with the following fluorescently conjugated monoclonal antibodies: CD8-QDOT®605 and CD4-PE-Texas Red® (Invitrogen); CD3-V450, PD-1 Alexa Fluor ®647, CCR5-PE-Cy™5, CD38-PE and HLA-DRFITC (BD Biosciences, San Jose, CA, USA). Cells were then fixed in 0.5% formaldehyde and data were acquired on a BD LSR II Flow cytometer (BD Biosciences), with ≥ 200,000 lymphocytes collected for each sample. Data were compensated and analyzed in FlowJo V9 (TreeStar, Ashland, OR). Populations of CD3^+^CD4^+^ and CD3^+^ CD8^+^ T cells were defined after standard, lymphocyte, singlet, and dead cell exclusion gates were applied to the data. Fluorescent-minus-one (FMO) controls were used to define positive gates for expression of CD38, CCR5, HLA-DR, and PD-1 on CD3^+^CD8^+^ and CD3^+^CD4^+^ T-cell populations.

The frequency of HIV-specific T cells, in PBMC and isolated colorectal mucosal cells, was measured by CFC as previously described [30, 31]. Briefly, thawed and rested PBMCs and freshly-isolated colorectal mucosal cells were stimulated with HIV-1 SF2 GAG overlapping peptide pools in the presence of Brefeldin A, monensin (Sigma-Aldrich), and CD107a-PECY5 (BD Bioscience). Following stimulation, cells were treated with EDTA, washed in PBS and stained with LIVE/ DEAD® Fixable Aqua Dead Cell Stain Kit as described above. The PBMCS were then fixed and permeabilized in FACS lyse and FACS Perm (both from BD Biosciences), and colorectal mucosal cells were fixed and permeabilized with BD Cytofix/Cytoperm (BD Bioscience). Cells were then stained with fluorescently labeled monoclonal antibodies CD3, CD4 and CD8 (described above) and IFN-γ-FITC, IL-2-PE, and TNFα-Alexa Fluor 700 (all BD Bioscience). Data from 500,000 lymphocytes were acquired, compensated and analyzed as described above to define CD3^+^CD4^+^ and CD3^+^CD8^+^ T-cell populations. The FMO controls (using CD3-stimulated cells) and no-stimulation controls were used to define positive gates for each of the individual functional markers on both T-cell populations and used to determine the frequency of each individual marker. The Boolean function in FlowJo was then used to calculate the frequency of each of the 16 possible combinations of these functional markers on each T-cell population.

### IMMUNOHISTOCHEMISTRY AND QUANTITATIVE IMAGE ANALYSIS

Immunohistochemistry was performed on 5 μm rectal biopsy tissue sections mounted on Superfrost Plus Microscope Slides (Fisher Scientific), which were dewaxed and rehydrated with double-distilled H_2_O (ddH_2_O). Heat-induced epitope retrieval (HIER) was performed by heating sections in 0.01% citraconic anhydride (containing 0.05% Tween-20), 1x Diva buffer (Biocare Medical), or Tris pH 8.6 solution (10mM Tris-HCL, 30mM NaCl and 0.025% Tween-20) in a pressure cooker (Biocare Medical) set at 122°C–125°C for 30 seconds. For samples stained for collagen 1 and Phospo-Smad3, after cooling, the slides were rinsed in ddH_2_O and then incubated for 10–20 minutes at room temperature in 20mM Tris-HCL containing 2mM CaCI_2_ and protein-ase K (2.0–4.0 μg/ml). Slides were incubated with blocking buffer (TBS with 0.05% Tween-20 and 0.25% casein) for 10 minutes. For CD4^+^ T cell IHC, slides were incubated with mouse anti-CD68 (1:400; clone KP1, Dako), mouse anti-CD163 (1:400; clone 10D6; Novocastra/Leica) and rabbit monoclonal anti-CD4 (1:200; clone EPR6855; Abcam, Inc.) diluted in blocking buffer over night at 4°C. Slides were washed in 1X TBS with 0.025% Tween-20 and endogenous peroxidases blocked using 1.5% (v/v) H_2_O_2_ in 1X TBS (pH 7.4) for 10 minutes. Slides were incubated with Mouse Polink-1 AP followed by Rabbit Polink-1 HRP for 30 minutes at room temperature. Sections were first incubated with Impact™ DAB (3,3′-diaminobenzidine; Vector Laboratories) to develop the CD4, washed and developed with Warp Red (Biocare Medical, Inc.) to mask the faint CD4 expressed on APCs and allowing for specific identification of CD4^+^ T cells. For collagen 1, myeloperoxidase (MPO), and Phospo-Smad3 IHC, slides were incubated with mouse monoclonal anti-collagen Type I (Sigma; clone: COL-1) diluted 1:800, rabbit polyclonal anti-myeloperoxidase (Dako; cat. no. A0398) diluted 1:1,000, or rabbit monoclonal anti-Phospo-Smad3 (Epitomics; pS423/425; cat. No. 1880-1) diluted 1:5,000, and all were incubated for 60 minutes at room temperature. Detection was performed using Mouse Polink-2 HRP for collagen 1, Rabbit Polink-1 HRP for MPO, or Rabbit Polink-2 HRP for P-Smad3 (All GBI Labs) according to the manufacturer's instructions. Sections were incubated with Impact™ DAB (3,3′-diaminobenzidine; Vector Laboratories) for 2–8 minutes, slides washed in ddH_2_O, counterstained with hematoxylin, mounted in Permount (Fisher Scientific), and scanned at high magnification (400×) using the Aperio AT2 System (Leica Biosystems) yielding high-resolution data for the entire tissue section. Representative regions of interest (ROIs; 0.04 mm^2^) were identified and high-resolution images extracted from these whole-tissue scans. The percentage of the area of the lamina propria that stained for CD4^+^ T cells (excluding APC CD4), collagen 1, myeloperoxidase (MPO) and Phospho-Smad3 was quantified using Photoshop (CS5 and CS6; Adobe Systems), Noiseware 5 (Imagenomic) for noise reduction, and Fovea Pro 4 (Reindeer Graphics) image analysis tools. In brief, high resolution ROI images were analyzed by segmenting the positive area with color channel thresholding in Photoshop, and measuring the positive area percentage with Fovea. For collagen 1, myeloperoxidase, and Phospho-Smad3 ROIs, stained with DAB, images were segmented by thresholding the CMYK yellow color channel and quantified as previously described [38]. Quantification of CD4^+^ T cells was performed as previously described [50] on slides stained for CD4 with DAB and myeloid cells (CD68+ CD163+) stained with Warp Red. Segmenting ROIs with both DAB and Warp Red staining required additional processing to avoid interference between the 2 stains, since they overlap in our analysis method. First, the Warp Red staining was masked by duplicating the image; converting it to the Lab color mode and binarizing color channel with a threshold chosen so that the Warp Red-stained areas became white, and the remaining area was black; pasting the binarized image as a new layer in the original image, and applying the “Lighter Color” layer blending mode masked Warp Red staining. Next, DAB-positive areas were measured by thresholding the CMYK yellow color channel as above. Then, we measured Warp Red staining; the masking layer was duplicated and inverted, making a new layer where the Warp Red-positive staining was black; the positive area percentage was measured as usual. This approach allows for specific quantification of both CD4^+^ T cells and myeloid lineage cells.

**Supplementary Table.** Correlations between markers of Monocyte Activation and Demographic and Clinical variables

Variable	rho	*P*-value
Age		
D dimer	**0.4672**	**0.0092**
IL-6	0.2564	0.1714
sCD14	0.1274	0.5024
STNF-R1	0.319	0.0858
sTNF-R2	**0.4568**	**0.0112**
HA	**0.6522**	**0.0001**

CD4 Baseline		
D dimer	−0.0231	0.9034
IL-6	**−0.4613**	**0.0103**
sCD14	−0.1733	0.3597
TNF-R1	**−0.382**	**0.0372**
TNF-R2	−0.3063	0.0997
HA	−0.077	0.6859

CD4CD38DR		
D dimer	0.1493	0.431
IL-6	**0.4279**	**0.0183**
sCD14	−0.0105	0.9563
TNF-R1	**0.5226**	**0.0031**
TNF-R2	**0.4932**	**0.0056**
HA	−0.0151	0.9368

CD8CD38DR		
D dimer	0.3803	0.3820
IL-6	0.3347	0.0706
sCD14	−0.0783	0.6808
TNF-R1	**0.4385**	**0.0153**
TNF-R2	**0.5191**	**0.0033**
HA	0.2114	0.2621

CD4PD1		
D dimer	0.1108	0.5599
IL-6	**0.574**	**0.0005**
sCD14	0.3293	0.0756
TNF-R1	**0.5491**	**0.0017**
TNF-R2	**0.3945**	**0.031**
HA	−0.1591	0.401

CD8PD1		
D dimer	0.6291	0.6522
IL-6	0.1438	0.4483
sCD14	0.3602	0.0505
TNF-R1	**0.3693**	**0.0446**
TNF-R2	0.0986	0.6043
HA	0.0897	0.6375

Correlations with *P*-values of < 0.05 are in bold
